# Socioeconomic inequalities in health-related fitness gradient shifts between 2001 and 2022 in young Polish adults

**DOI:** 10.3389/fpubh.2023.1163215

**Published:** 2023-05-09

**Authors:** Jarosław Domaradzki, Dawid Koźlenia, Katarzyna Kochan-Jacheć, Paweł Szkudlarek, Jarosław Fugiel

**Affiliations:** Unit of Biostructure, Faculty of Physical Education and Sport, Wroclaw University of Health and Sport Sciences, Wroclaw, Poland

**Keywords:** health inequalities, health-related fitness, young adults (18–25 years old), socio-economic factors, body morphology, physical fitness

## Abstract

**Background:**

This study aimed to assess the importance of socioeconomic status (SES) on health-related fitness (H-RF) measurements in young adults and determine the impact of SES over 20 years of substantial social and economic changes in Poland.

**Material and methods:**

The study compared H-RF differences between 2001 (P_1_) and 2022 (P_2_) in 252 volunteers aged 18 to 28 years who were grouped into quartiles based on SES and gender. The variables measured included height, weight, body mass index, body fat mass, hand strength (hand grip), abdomen strength (sit-ups), flexibility (sit and reach), and leg power (standing long jump), with a synthetic motor performance index (MPSI) calculated for each participant.

**Results:**

Health-related differences based on social inequalities included body fat mass and MPSI, and two-way analysis of variance (ANOVA) revealed an interaction between SES and period on motor performance (F = 2.73, *p* = 0.045). In addition, *post-hoc* tests revealed differences in P_1_ between SES quartiles one and two (*p* = 0.028). Over the last 20 years, physical fitness decreased and body fat increased. The regression slope showed decreased motor performance with higher amounts of body fat in P_2_ subjects compared to their P_1_ peers.

**Conclusion:**

The observed trends may be associated with lifestyle changes shaped by technology development, high-energy and low-quality food access, and increased physical inactivity.

## 1. Introduction

Socioeconomic status (SES) is defined by income, educational level, family structure, and degree of urbanization, among other factors, and its relationship with health is researched widely for numerous public health reasons. Among the many health-related impacts of SES, including its psychological, mental, physical, and physiological effects, indicators of physical fitness are rarely used (1–3). Indeed, an increasing number of studies have focused on physical activity rather than physical fitness, especially health-related fitness (H-RF) (4–6). Health outcomes mirror differences in socioeconomic position (lower–higher), defined as socioeconomic inequalities in health (7). Health inequalities are “systematic differences in the health of people occupying unequal positions in society” and are primarily “unjust and avoidable.” They are complex and observed in many different, often overlapping, social dimensions such as income, social class, geography, ethnicity, disability, and gender. Some of these dimensions can be ranked, such as income, and some cannot, including ethnicity (8, 9). Thus, health inequalities are systematic differences in the health status of different socioeconomic groups, and several health-related variables reflect changes in socioeconomic conditions over a long period (balancing or deepening of the differences). Several studies demonstrated, amid a global pandemic and continued rise in obesity, that the gap in inequalities is being widened further (10).

One description of health in terms of public health is physical fitness directly related to health, referred to as H-RF (11). In this regard, systematic physical activity directly impacts health and H-RF (12, 13). H-RF includes a combination of abdominal strength and endurance, lower back and upper thigh flexibility, cardiorespiratory fitness, and body composition (11), and a lack of physical activity leads to an excessive increase in body fat (14). Recent H-RF definitions also include metabolic and body morphology components (15). Increasing H-RF by improving cardiovascular and muscular functioning, for example, and focusing on body composition quality, leads to improved health (16). Such improvements directly translate into reductions in the development of cardiovascular diseases, diabetes, and hypertension. They also improve emotional control, reduce stress, and help maintain an appropriate level of body weight, which is one of the components of H-RF (12, 17–19). Biological traits that are health measures, broadly understood as H-RF, that respond to environmental changes such as economic and social factors, are a sensitive “barometer” of the economic and social situation of groups of people (20).

Those who occupy low positions in the social hierarchy are estimated to have at least twice the risk of severe illness and premature death (21–23). The negative impact of low SES on health is explained by its association with an unhealthy lifestyle, higher exposure to stress, low level of social support, lower level of health knowledge, and limited access to health services, especially specialist services (24–27). Moreover, growing up in impoverished and marginalized socioeconomic conditions shortens life expectancy and contributes to poor mental and physical health (28–30).

Health inequalities are measured using a variety of procedures and methods. Recognized measures of socioeconomic health inequalities include, among others, the slope of inequality index (SII) and the relative inequality index (RII) (31, 32). Many national and international programs focus on reducing social inequalities in countries and social groups (33, 34). When measuring differences between more and less privileged groups, the results depend on the factors assessed (34). In a study by Hauspie et al. (35), the secular trend in the achieved height and growth rate was more pronounced in children from lower socioeconomic backgrounds, such as those from low-educated families and rural areas. However, the results of many studies indicate a lack of clear assessment of the impact of SES on the existence or absence of social inequalities in health (36). Furthermore, few studies have focused on the differences between the least advantaged and most advantaged groups or regions because they aimed to merely document the existence of these differences (34).

Health inequities tend to change over long periods, such as 10, 20, or 50 years (37). According to the literature, no studies have examined if or how the impact of social differences has changed H-RF components. In Poland, there have been substantial economic, educational, and cultural changes in the last 20 years, which may be the background for changes in social inequalities that impact H-RF. Moreover, restrictions imposed during the coronavirus disease (COVID-19) pandemic have left their mark. In this regard, ongoing health monitoring in the context of H-RF is necessary, and a comparison of today's population to the same from 20 years ago will allow for the assessment of the directions of changes in H-RF in young adults from families with different economic and social statuses.

Since anthropometrical and functional features are health markers, social gradients in health, whereby morpho-functional features improve as SES rises, can be used to assess health inequalities of different subpopulations or groups of people. Indeed, foundational knowledge of the reciprocal interrelations between secular changes and social gradients is crucial in determining the nature of health inequalities and the scale of changes over time, highlighting the groups of young adults most at risk and identifying features that most strongly reflect environmental changes. The problems of intergenerational changes were often undertaken but were addressed to average populations. Only a few studies assessed secular trends concerning young people who are athletic or are engaged in physical activity, such as Physical Education and Sport Faculty students (38). To the best of the authors' knowledge, no studies have assessed secular trends in health-related fitness (H-RF) in such physically active subpopulations. Furthermore, there are no studies focused on the secular trends in H-RF from a social-economic inequalities perspective. Therefore, this study aimed to assess the importance of SES on H-RF measurements in young adults and how the impact of SES changed over 20 years of substantial social and economic change in Poland by posing the following four questions: (1) Has the proportion of people with different economic and social statuses changed over the 20 years analyzed, and how has it changed? (2) Which variables reflect social inequalities, and were the variables the same in 2001 and 2022? (3) What were the absolute differences, measured using the SII, between those in the lowest and highest SES positions at the beginning of the 21^st^ century and 20 years later? and (4) Have these differences between the groups at either extreme changed over time, based on the RII?

## 2. Material and methods

This study follows up on the issues presented in the study by Fugiel et al. (1), which provides a detailed description of the methodology, test subjects, and procedures. Here, the most important aspects are briefly described.

### 2.1. Ethics

The Senate Research Ethics Committee of the Wroclaw University of Health and Sport Sciences in Poland granted permission for this research, which followed the guidelines of the Declaration of Helsinki (consent number 16/2018). All subjects gave written voluntary consent to participate in the project before starting the study. Participants received detailed information about the research purpose, type, and methods and could withdraw from the research at any time without giving a reason.

### 2.2. Participants/sample

Two groups of students provided measurement data, with results obtained in March 2001 (P_1_) and March 2022 (P_2_). Participants were students recruited from the Faculty of Physical Education and Sport, with employees from the Department of Biostructure of the Wroclaw University of Health and Sport Sciences carrying out the research. Participants (*n* = 252, aged 18 to 28 years) comprised 120 men (M) and 127 women (F), with 70 M and 78 F included in 2001 and 50 M and 49 F examined in 2022. All subjects declared no participation in professional sports. The additional inclusion criterion was good health declared in the questionnaire and lack of musculoskeletal injuries 2 weeks before studies. The exclusion criterion was hospitalization during the semester the studies were conducted.

### 2.3. Procedures

The research was conducted at the Biokinetics Research Laboratory of the Wroclaw University of Health and Sport Science, which has the Polish Standard-European Standard International Organization for Standardization (PN-EN ISO) 9001:2009 Quality Management System Certificate (certificate reg. no. PW-48606-10E). Testing occurred in the morning, and all subjects were to refrain from eating, drinking, and exercising for at least 3 h before testing. Measurements of somatic features and physical fitness followed established procedures.

### 2.4. Measurements

#### 2.4.1. Somatic features

Trained personnel performed each anthropometric measurement privately in a separate room. Body height (BH) and body weight (BW) measurements followed the procedures of Martin and Saller (39). BH was measured with an accuracy of 0.1 cm using a Swiss Anthropometer (GPM Anthropological Instruments, DKSH Ltd, Switzerland). BW was measured to the nearest 0.1 kg using a SECA M799 (type approval D07-09-032, Hamburg, Germany).

The Harpenden Skinfold Caliper measured skin folds of the triceps brachii and abdominal muscles with an accuracy of 0.1 mm (1). Body fat percentage (BFP) was calculated from skin fold measurements using the equations of Slaughter et al. (40) for comparative analysis.

#### 2.4.2. Physical fitness of the musculoskeletal system

Four tests (Eurofit) assessed physical fitness, including hand strength (HS), abdomen strength (ABS), flexibility (Flex), and leg power (SJ). HS tests measured hand grip strength with an accuracy of 1 kg using a JAMAR hydraulic hand dynamometer (Sammons Preston Rolyan, IL, USA) with an adjustable handle set in position two. Analysis of HS results used the best score from two trials. Meanwhile, the number of full sit-ups performed within 30 s (timed sit-ups) determined ABS from one trial, during which participants adopted a sitting position and reached as far forward as they could along a measurement line with one hand placed on top of the other. A standing long jump, measured as the distance from the measurement line to the heel closest to the measurement line at the maximum jump, determined SJ, with the best score from two trials used for analysis. All data were standardized using the formula,


$$\begin{array}{l} z_{ij}=\frac{x_{ij}-{\bar{x}}_{j}}{s_{j}}, \end{array}$$

where z_**ij**_ is the standardized value, x_ij_ is the j-variable of the i-object, $\bar{\text{x}}$ is the mean value of the j-variable, and s_j_ is the standard deviation (SD) of the j-variable (41).

After transformation, the diagnostic variables were standardized between 0 and 1, which made it possible to compare and estimate patterns and the distance from them (41).

Body mass index (BMI), BH, and BFP were assessed as single variables. Secular trends in motor function and social-economic gradients were related to general motor performance. Therefore, these variables were combined into a motor performance synthetic index (MPSI) using multidimensional comparative analysis (MCA), which uses a different scale and units. MCA analysis adopted the Hellwig (42) pattern of development method, for which a detailed description of the steps used in its design was published by Hellwig (42).

#### 2.4.3. Socioeconomic status

The SES assessment was based on five socioeconomic factors, including urbanization level (city, town, or village, with a higher urbanization level equating to a higher SES), family size (one, two, three, four, or more children, with a smaller family resulting in a higher SES), parents' education level (elementary school, secondary school, trade school, or university, with a higher education level leading to a higher SES), and family type during the students time at the family home as a child (one or two parent households as no students lived without parents, and two-parent households led to a higher SES).

The first factor of principal component analysis (PCA) defined the general SES variable. The first-factor eigenvalue for P_1_ was 1.61, which explained 32.24% of the variance, and factor loadings were 0.51–0.37. For P_2_, the eigenvalue of the first factor was 1.58, which explained 31.60% of the variance, with factor loadings of 0.77–0.42.

Division of SES values into quartiles resulted in four categories, very low, low, medium, and high. Mean values calculated for the indices of each category underwent regression analysis to calculate health inequality indices.

### 2.5. Statistical analysis

All calculations used Statistica 13.0 software (StatSoft Poland, Cracow, Poland). The student numbers in each SES category were presented as percentages, accumulated proportions, and ranks. Pearson's chi-squared (χ^2^) test determined the statistical significance between the differences in expected and observed SES frequencies (43). The Shapiro–Wilk test evaluated the normality of the distribution of continuous variables. Descriptive statistics for continuous measurements (ABS, age, BFP, BH, BMI, BW, FL, HS, and SJ) and secular trends were published previously (1). The current study calculated and presented standardized BFP, BH, BMI, and MPSI for each period (P_1_ and P_2_) as mean and SD, with 95% confidence intervals (CI).

A two-way analysis of variance (ANOVA) assessed the differences between mean values of the anthropometric measurements and MPSI at the time (two levels: P_1_ and P_2_) and SES (four quartiles: very low, low, medium, and high) levels. *Post-hoc* tests made detailed comparisons between quartiles in each period and matching quartiles from the different periods.

Regression methods assessed inequalities in health using the calculated SII and RII indices. The SII and RII indices are regression-based indicators (8, 11) that rely on a regression relating to health outcomes of social groups relative to their position on the SES distribution. In the classic approach, the absolute inequality index (SII) is a linear regression coefficient based on data defining the health status index for each socioeconomic group and a variable determining the rank of this group based on social status (44). The results are interpreted as the absolute difference in health between the lowest-ranked social group and the social group with the highest rank in the adopted classification. Analyses of changes over time and comparisons use the RII, which is the absolute measure (SII) in relation to the mean health status of the population (45). This index is interpreted as a percentage difference in the health measures of groups at either end of the social hierarchy in relation to the mean level of health observed in the population. The slope of the regression line is the SII, a measure of absolute inequality, and is given by:


(1) $$\begin{array}{l} SH=\frac{\sum_{i=1}^{n}w_{i}\left(y_{i}-{ȳ}_{w}\right)\left(x_{i}-{\bar{x}}_{w}\right)}{\sum_{i=1}^{n}w_{i}{\left(x_{i}-{\bar{x}}_{w}\right)}^{2}} \end{array}$$

Where *x*_*i*_ is the ridit, *y*_*i*_ the mortality rate, and *w*_*i*_ the frequency of each class *i* = {1, …, *n*}, and ${\bar{x}}_{w}$ and ȳ_*w*_ the frequency-weighted averages of *x*_*i*_ and *y*_*i*_.

The RII is obtained by extrapolating the regression line toward the extreme (theoretical) positions of the x-axis, 0 and 1. It is calculated as a ratio of the value at the bottom of the social hierarchy (corresponding to the intercept) and the value at the top of the hierarchy (corresponding to the intercept + slope). RII is given by:


$$RII=\frac{Intercept}{Intercept+\;Slope},$$

The significance level was set to α = 0.05.

## 3. Results

Among all participating students, the majority (58.7%) were surveyed in P_1_, with 41.3% surveyed in P_2_. These unequal numbers reflect the typical decline in the number of students, which is related to, among other factors, a demographic decline and a lower recruitment rate.

The group with the lowest economic and social status (Q_1_) included 28% of people surveyed in P_1_ and 26% in P_2_. The groups with average family SES conditions included 30% (Q_2_) and 21% (Q_3_) of the respondents in P_1_ and 20% (Q2) and 29% (Q3) of the respondents in P_2_. Students with the highest family SES (Q_4_) comprised 22% of P_1_ and 25% of P_2_. These percentages indicate similar frequencies of people with the lowest SES in both periods, while the proportion of people in the subgroups with the highest SES (Q_3_ and Q_4_) increased in P_2_ (54%) compared to P_1_ (43%) (Table 1). However, the χ^2^ analysis did not show statistically significant differences in the proportions of people from individual quartiles in periods P_1_ and P_2_ (χ^2^ = 4.10, *p* = 0.251).

**Table 1:** The numbers, percentages, and cumulative proportions of the students by socioeconomic status groups (Q_1_-Q_4_) and period of examination (2001–2022).

		**Year 2001**			**Year 2022**	
**SES group**	**N**	**Cumulative proportion**	**Rank**	**N**	**Cumulative proportion**	**Rank**
Q1	41	0.277	0.139	27	0.260	0.130
Q2	44	0.574	0.462	21	0.462	0.361
Q3	31	0.783	0.679	30	0.750	0.606
Q4	32	1.000	0.892	26	1.000	0.875
Total	148			104		

Table 2 details the statistical characteristics of the normalized BH, body mass synthetic index (BMSI), and MPSI values.

**Table 2:** Basic statistics of the standardized morphological features and motor performance synthetic index (MPSI) of the students by socioeconomic status groups (Q1–Q4) and period of examination (2001–2022).

	**Year**		**2001**					**Year 2022**								
**SES group**	**Body height**		**BMI**		**Body fat %**		**Motor performance synthetic index**		**Body height**		**BMI**		**Body fat %**		**Motor performance synthetic index**	
	**Mean** ±**sd 95%CI**		**Mean** ±**sd 95%CI**		**Mean** ±**sd 95%CI**		**Mean** ±**sd 95%CI**		**Mean** ±**sd 95%CI**		**Mean** ±**sd 95%CI**		**Mean** ±**sd 95%CI**		**Mean** ±**sd 95%CI**	
Q_1_	0.14	1.00	0.08	1.01	−0.05	1.06	0.52	0.13	−0.11	0.86	−0.24	0.79	−0.20	0.66	0.51	0.12
	−0.18	0.45	−0.24	0.39	−0.39	0.28	0.48	0.56	−0.45	0.23	−0.55	0.08	−0.46	0.06	0.47	0.56
Q_2_	−0.04	1.09	−0.17	1.05	−0.01	1.02	0.53	0.13	−0.36	1.13	−0.26	1.12	−0.05	0.79	0.46	0.10
	−0.38	0.29	−0.49	0.15	−0.32	0.30	0.49	0.57	−0.88	0.15	−0.76	0.25	−0.40	0.31	0.42	0.51
Q_3_	0.10	1.00	0.09	0.98	0.05	1.01	0.46	0.11	0.32	0.95	0.28	0.91	0.02	1.21	0.53	0.13
	−0.26	0.47	−0.27	0.45	−0.32	0.42	0.42	0.50	−0.04	0.67	−0.06	0.62	−0.43	0.47	0.48	0.58
Q_4_	−0.22	0.84	0.05	0.94	0.04	0.91	0.52	0.16	0.04	1.00	0.13	1.11	0.22	1.16	0.38	0.50
	−0.52	0.09	−0.29	0.39	−0.29	0.36	0.46	0.58	−0.36	0.45	−0.32	0.58	−0.25	0.69	0.44	0.55

The two-way ANOVA of normalized values of all variables suggested no significant influence of either SES (BH: F = 1.86, *p* = 0.138; BMI: F = 1.87, *p* = 0.134; BFP: F = 0.70, *p* = 0.552; MPSI: F = 0.34, *p* = 0.795) or time (F = 0.03, *p* = 0.852; F = 0.06, *p* = 0.803; F = 0.02, *p* = 0.961; F = 0.28, *p* = 0.599). However, a significant interaction was observed between both factors in motor performance (F = 2.73, *p* = 0.045), which indicated that the change in SES impacted motor fitness over time.

The two-way ANOVA results justified detailed *post-hoc* comparisons of the quartiles within and between the periods. Within P_2_, BH differed significantly between Q_2_ and Q_3_ (*p* = 0.035), though BFP and BMI were similar between the quartiles. However, differences in MPSI between Q_2_ and Q_3_ were significant in P_1_ (*p* = 0.028). Comparisons of the same quartiles between the periods showed significant MPSI differences in Q_2_.

The primary analysis concerned the assessment of differences in the intensity of the relationship between SES position and morphological and motor variables over time. The regression models analyzed SII and RII indices of inequalities, where the β1 statistic was the absolute index (SII) (see results in Table 3).

**Table 3:** The results of the linear regression models estimation.

**Period**	**Independent variable**	**β_1_**	**SE (β_1_)**	**t**	**p**	**RII**
**BH (normalized)**						
2001	Constant	0.18	0.15	1.23	0.343	−1.085
	Rank	−0.35	0.25	−1.43	0.290	
2022	Constant	−0.25	0.30	−0.83	0.494	−1.250
	Rank	0.45	0.54	0.85	0.487	
**BMI**						
2001	Constant	−0.02	0.16	−0.13	0.908	−0.527
	Rank	0.06	0.26	0.23	0.840	
2022	Constant	−0.34	0.21	−1.65	0.240	−1.104
	Rank	0.65	0.37	1.78	0.216	
**BFP**						
2001	Constant	−0.07	0.02	−2.93	0.099	−0.981
	Rank	0.13	0.04	3.57	0.070	
2022	Constant	−0.27	0.04	−7.41	0.018	−0.980
	Rank	0.54	0.06	8.47	0.014	
**MPSI**						
2001	Constant	0.52	0.04	13.76	0.005	1.057
	Rank	−0.03	0.06	−0.45	0.700	
2022	Constant	0.50	0.04	13.61	0.005	1.003
	Rank	0.00	0.07	−0.02	0.984	

The lack of significance in BH and BMI indicated no inequalities in these health markers. Indeed, similar patterns emerged in the relationship between anthropometric features and the socioeconomic position of P_1_ and P_2_ participants (Table 3).

**Figure 1:**
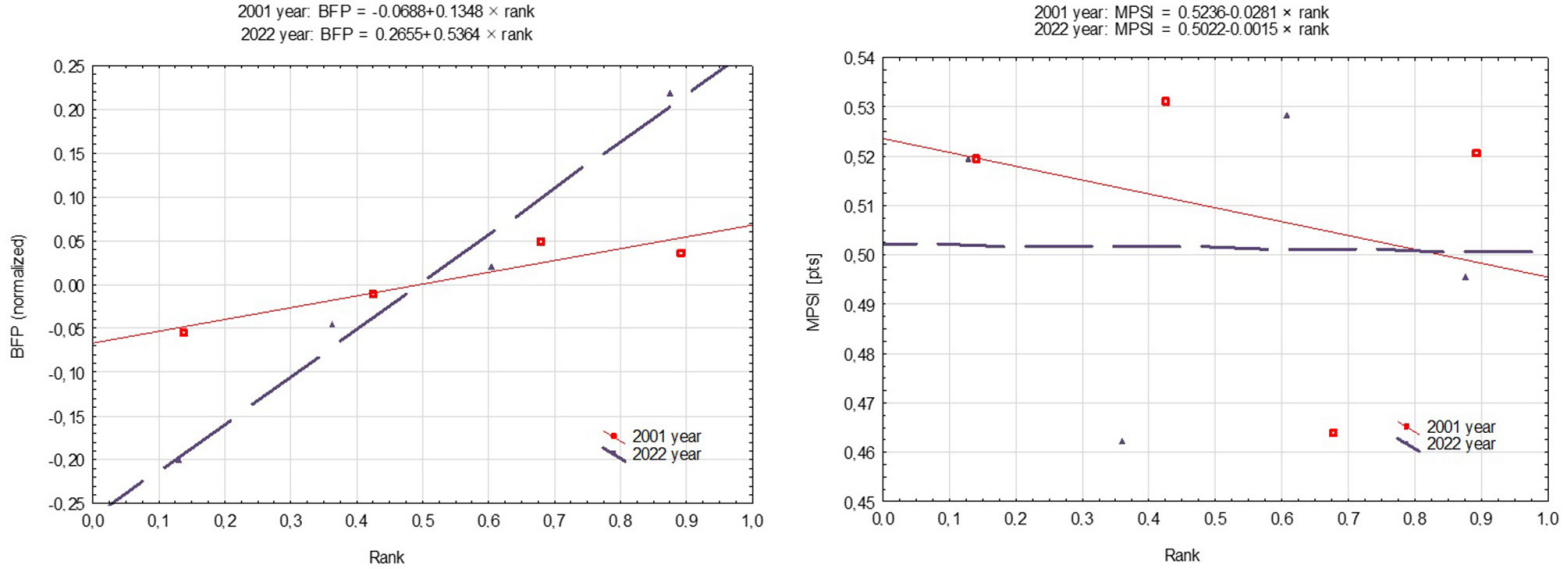
Health inequalities in students by the period of examination using a regression-based approach.

BFP increased along the SES axis, with lower values in those with the worst living conditions and higher values in those living under the best conditions (Figure 1; Table 3). The BFP regression for the participants examined in P_2_ was steeper, which suggests a more intense effect of social inequalities on BFP in P_2_ than in P_1_. Interestingly, BFP in students with lower SES (Q_1_-Q_2_) in P_1_ was higher than in Q_1_ and Q_2_ from P_2_, though the opposite was true for Q_3_ and Q_4_. This indicates a deeper gap between SES groups examined in 2022 and an intensification of the effects of inequalities on BFP. In contrast, the motor performance gradient had the steepest decline along the SES axis, from lowest (Q_1_) to highest (Q_4_), in P_1_. As such, P_2_ students in Q_1_-Q_3_ had lower motor performance levels. However, P_2_ Q_4_ students had lower MPSI levels than P_1_, with all student scores identical.

The absolute inequality indicators confirmed social inequalities at the BFP level related to the socioeconomic position of student families. There are greater differences in health now (P_2_ SII = −0.27) than 20 years ago (P_1_ SII = −0.07). The RII shows that the differences in the level of BFP between people in the highest (Q_4_) and the lowest (Q_1_) socioeconomic positions were at 27% in P_2_ and 7% in P_1_. Regarding MPSI, the intensity of differences between extreme SES groups was 2% higher in P_1_ (SII = 0.52) than in P_2_ (SII = 0.50).

RII illustrates the relationship between health markers and SES grouping in relation to the mean level of these markers observed in the studied groups. For BFP, the RII indicators were practically identical, indicating that the same proportion of people in the highest SES from P_1_ and P_2_ had a higher level of adiposity than the mean group score. MPSI was the same across SES groups in P_2_ (RII = 1), although scores in Q_4_ were 6% lower than the mean level of motor performance in the entire group of respondents.

## 4. Discussion

This study demonstrated SES-related changes to motor performance and body composition over 20 years. The last two decades have seen substantial lifestyle changes associated with technology development (46). However, social inequalities still strongly affect health features, as the risk of being overweight increases by 2-fold. In this regard, higher SES equated to higher body fat indicators, which could lead to health-related problems associated with metabolic disorders and cardiovascular diseases (47).

A declining trend in motor performance levels was evident 20 years ago, which seems to be constant in current times. Furthermore, a comparison of the groups from both periods indicated a significant worsening in motor performance. These data provide deeper insight into H-RF changes and indicate the need for public health disease prevention interventions (48). The decline in motor performance requires attention because motor skills are lower at a young age, meaning future generations are at risk of limited independence in old age (1, 49). Moreover, this creates a severe public health issue that could grow if the tendency develops.

A study by Baran et al. (50) among school-aged children showed a tendency of increased BMI in subjects living outside the city. This was due to a lack of health-related knowledge, limited access to good-quality food, and the limited availability of leisure activities in smaller towns. Nonetheless, family is the primary factor affecting health-related inequalities.

Health inequalities were less visible in Swedish student populations in periods similar to this study (51), indicating that political and economic states strongly affect citizens' lives. Dalstra et al. (52) showed a higher likelihood of cardiovascular issues in lower SES groups, which could be related to inequalities in access to health services and early diagnosis. Furthermore, health inequalities pose a substantial risk of life quality decline and can be a source of many mental diseases and disorders (53). However, a healthy lifestyle is vital to disease prevention, irrespective of SES differences, and this is worsening in groups with limited access to health services (54). This growing issue became apparent throughout the recent COVID-19 pandemic, during which the risk of adverse disease was higher in individuals with low SES and worse lifestyles (55).

A decline in physical activity is linked to increased body fat (56), and a sedentary lifestyle combined with an unhealthy diet leads to many diseases of civilization (57). Indeed, physical activity is one of the most crucial preventive actions not strongly related to SES. One of the reasons for growing physical inactivity is the development of technologies (46). Despite the many advantages associated with the development of numerous forms of transport and communications, and the possibilities provided by the internet, people need to do less in their daily routines, which means they move less and expend less energy. In addition, inactivity is prolonged due to the many hours of leisure time now spent using phones or computers (58). These factors all translate to growing body fat and a decline in motor performance, which was also visible in the current study. It was shown that low health-related fitness level in youth is associated with cardiometabolic risk (59). Therefore, there is a need to popularize many forms of physical activity from school age (60), which should decrease the risk of many health problems or independency in older years (61, 62).

We are aware of some limitations in the current study. The study was conducted on a select group of students from a single institution, which limits the applicability of the findings to a larger population. A more extensive and diverse sample is needed to provide a comprehensive understanding of health inequalities in the overall population of Poland. The absence of body fat data from 2010 and 2011 limited the Slaughter regression analysis and would have provided a deeper understanding of the observed trends. On the other hand, the study presented unique data on the association between SES, body morphology, and motor performance. This data provide insight into the changes associated with H-RF factors and fills a gap in the literature. Another limitation is the lack of several different potential confounding variables such as lifestyle (smoking and alcohol consumption) or dietary behaviors. In addition, more socioeconomic factors (e.g., occupation, income, and wealth) could be used to assess a family's socioeconomic situation.

## 5. Conclusion

The regression method provided information on the intensity of inequalities in health by calculating the RII, which determined the relative disadvantage experienced by SES subgroups over 20 years. This method better reflected the relationship between the economic and social living conditions of families (SES) and the variables characterizing health than comparisons of means using analysis of variance. Furthermore, the juxtaposition of the two periods made it possible to compare the intensity of social differences in health.

Time did not significantly differentiate the overall percentage of people from individual SES groups, though it affected the social gradient concerning BFP, which was much steeper in P_2_ than in P_1_. However, a slight increase in BFP was evident in higher SES subgroups, which relates to strong economic development.

Adipose tissue was and is a sensitive barometer of economic and social status, even within a select group of physical activity students from the University of Physical Education. The increase in the dependence of BW and its fat component on the SES position (between Q_1_ and Q_4_) in P_2_ may be the result of strong technological development in the last two decades. Such changes have changed behavior patterns related to spending free time (more time in front of computers and televisions and less physical activity), traveling, and undertaking physical work.

A specific group at risk of excessive BFP are those from more affluent families, for whom improper nutrition may be connected to fast food, among other factors. On the other hand, the physical activity of the groups included in this study may equalize the level of physical fitness, regardless of SES. However, in terms of physical fitness level, students from the P_1_ period were characterized by a higher level, which could be due to a combination of a higher level of physical activity and changes in body morphology (adipose tissue). This finding suggests a strong relationship between motor fitness level and body morphology. The results presented indicate that changes in morphological and motor health indicator variables should be monitored periodically using the regression method described.

## Data availability statement

The raw data supporting the conclusions of this article will be made available by the authors, without undue reservation.

## Ethics statement

The studies involving human participants were reviewed and approved by the Senate Research Ethics Committee of the Wroclaw University of Health and Sport Sciences in Poland (consent number 16/2018). The patients/participants provided their written informed consent to participate in this study.

## Author contributions

JD conceived the study. JD, DK, KK-J, and PS collected data for the study. JD and JF analyzed the data. All authors were involved in drafting the article, reviewing, and approving the final manuscript.
